# Enhanced photoluminescence and shortened lifetime of DCJTB by photoinduced metal deposition on a ferroelectric lithography substrate

**DOI:** 10.1038/s41598-022-10303-y

**Published:** 2022-04-13

**Authors:** Yuan-Fong Chou Chau, Hao-En Chang, Po-Sheng Huang, Pin Chieh Wu, Chee Ming Lim, Li-Ming Chiang, Tzyy-Jiann Wang, Chung-Ting Chou Chao, Tsung Sheng Kao, Min-Hsiung Shih, Hai-Pang Chiang

**Affiliations:** 1 Centre for Advanced Material and Energy Sciences, Universiti Brunei Darussalam, Tungku Link, Gadong, BE1410 Brunei Darussalam; 2 Department of Optoelectronics and Materials Technology, National Taiwan Ocean University, Keelung, 202 Taiwan, ROC; 3 Department of Photonics, National Cheng Kung University, Tainan, 70101 Taiwan, ROC; 4 Department of Photonics & Institute of Electro-Optical Engineering, College of Electrical and Computer Engineering, National Chiao Tung University, Hsinchu, 300 Taiwan, ROC; 5 Institute of Electro-Optical Engineering, National Taipei University of Technology, Taipei, 10608 Taiwan, ROC; 6 Research Center for Applied Sciences, Academia Sinica, Taipei, 11529 Taiwan, ROC

**Keywords:** Nanoscience and technology, Optics and photonics, Physics

## Abstract

The photodeposition of metallic nanostructures onto ferroelectric surfaces could enable new applications based on the assembly of molecules and patterning local surface reactivity by enhancing surface field intensity. DCJTB (4-(dicyanomethylene)-2-t-butyl-6-(1,1,7,7-tetramethyljulolidyl-9-enyl)-4H-pyran) is an excellent fluorescent dye and dopant material with a high quantum efficiency used for OLED displays on the market. However, how to raise the photoluminescence (PL) and reduce the lifetime of DCJTB in a substrate remain extraordinary challenges for its application. Here, we demonstrate a tunable ferroelectric lithography plasmon-enhanced substrate to generate photo-reduced silver nanoparticles (AgNPs) and achieve enhanced PL with a shortened lifetime depending on the substrate’s annealing time. The enhanced PL with shortened lifetimes can attribute to the localized electromagnetic (EM) wave produced by the nanotextured AgNPs layers' surface and gap plasmon resonances. The simulation is based on the three-dimensional finite element method to explain the mechanism of experimental results. Since the absorption increases, the remarkable enhanced PL of DCJTB can attain in the fabricated periodically proton exchanged (PPE) lithium niobate (LiNbO_3_) substrate. Furthermore, the proposed fabrication method demonstrates to help tune the surface EM wave distribution in the substrate, which can simultaneously achieve the significantly shortened lifetime and high PL intensity of DCJTB in the substrate. Compared with the un-annealed substrate, the PL intensity of DCJTB in the assembly metallic nanostructures is enhanced 13.70 times, and the PL’s lifetime is reduced by 12.50%, respectively. Thus, the fabricated substrate can be a promising candidate, verifying chemically patterned ferroelectrics' satisfaction as a PL-active substrate.

## Introduction

The presence of metal nanoparticles (MNPs) arranged in nanostructure offers a strong light-matter interaction resulting in “hot spots” with intensely large electromagnetic (EM) waves due to the structure’s plasmonic effect^1–7^. The concentration of high EM wave in the plasmon-enhanced substrate can widely apply for surface-enhanced Raman scattering (SERS)^8–17^ and other applications in nanophotonics^18–21^, and notably in applications requiring high wavelength sensitivity and selectivity^22–24^. The fabrication of functional nanomaterials with different MNPs in the plasmon-enhanced substrate called photoluminescence (PL) substrates has attracted considerable attention^25–29^. Several fabrication techniques include lithography-based techniques, such as holographic lithography^30,31^, focused ion beam patterning^32,33^, direct imprint^34^, nanosphere lithography^35,36^, thermal dewetting^37^, electron beam lithography^38,39^ and neutral beam etching^40^. These methods can produce high-quality arrays of plasmon-enhanced substrates; however, the processes are highly time-consuming and expensive because of the equipment's capital cost. Their reproducibility rate can be low^41–44^.

Recently, ferroelectric materials, e.g., lithium niobate (LiNbO_3_), as templates for the "growth" of patterned or structured MNPs has received numerous attention. It drives by the increasing demand for plasmon-enhanced substrates in the fields of biochemical sensors or reconfigurable electronics^26,45–48^. The solitary characteristics of ferroelectric surfaces can obtain electrowetting^49,50^, nanostructured surface functionalization, and localized chemical reactions^51^, especially in the use of localized charge transfer oxidation–reduction chemical processes capable of producing nanostructures of MNPs on the surface of ferroelectric substrates^52^. These substrates can serve as functional substrates for congregating complex nanostructures that combine with their surface electronic features. MNP fabrication on LiNbO_3_ ferroelectric surfaces can process using a photochemical reaction^53^. Ferroelectric lithography is an emerging technology that sanctions directed self-assembly. This technique has the potential in cost-cutting of MNP arrays production^54^. One of the potential techniques utilizes congruent single-crystal LiNbO_3_ as the ferroelectric template^52^. LiNbO_3_ has the merit of a single 180°-domain structure and reveals a powerful photovoltaic effect along the + z direction, in which the photoinduced electrons can migrate adequately^55^. When an ultra-violet (UV) light (~ 4.89 eV) impinges on the LiNbO_3_ (bandgap ~ 3.9 eV), electron–hole pairs can produce in the LiNbO_3_. The periodically modulated spontaneous polarization separates the electrons and holes, and the electrostatic field accelerates the electrons (photoelectrons) to the proton exchanged region surface. In the presence of the AgNO_3_ solution, the silver (Ag) ions can also speed up by the electrostatic field to the surface of the proton exchanged region, and the photoelectrons then diminish them to form a silver nanoparticle (AgNP) array^56^. It is, therefore, easy to deposit on the substrate by only utilizing the photovoltaic effect. Ag deposition occurs at the domain boundaries^57^.

There are several methods in producing plasmon-enhanced substrates. The physical mechanism on the enhanced fluorescence and shorter lifetime has been interpreted by Chen et al^58^. Wang et al. explained the mechanism of the direction of polarization in the LiNbO_3_ substrate and elucidated the photoelectrons migrating to the substrate surface can reduce the Ag ions^59^. Rodriguez et al. proposed a chemical patterning method to prepare the periodical proton exchanged (PPE) LiNbO_3_^53^, and they claimed that the PPE LiNbO_3_ substrates have a stronger SERS signal due to the SPR effects^60^. However, the PPE LiNbO_3_ substrates reveal surface morphology in the range of 6–8 nm along the z-direction^61^, which could be a drawback for SERS application. Subsequently, Damm, S. et al. fabricated Ag nanopatterns using periodically poled LiNbO_3_ and periodically proton-explanted template methods^60^. Liu et al. reported on the fabrication of large-scale nanoparticle arrays SERS substrates based on periodically poled LiNbO_3_ templates^55^. Tseng et al. proposed a template with color surface plasmon-enhanced PL of organic dyes using annealed AgO_x_ thin Film^62^. Ferroelectric lithography potentially provides a route to fabricate substrates that offer reproducible plasmon-enhanced substrates made from the MNP arrays of AgNPs^53^. For the benefits of expediency and reproducibility, photochemically deposited MNPs on ferroelectric-based substrates have great potential in surface-plasmon-related applications^63^.

OLED (Organic light-emitting devices), as an exemplary flat panel display and solid-state lighting source, have attracted numerous considerations since their advantages are low cost, low consumption, and environment-friendly^64^. An archetypical dopant material used for many of today’s OLED displays on the market is DCJTB (4-(dicyanomethylene)-2-t-butyl-6-(1,1,7,7-tetramethyljulolidyl-9-enyl)-4H-pyran), which is an excellent red fluorescent dye with solution PL λ_max_ ~ 620 nm and a quantum efficiency > 90%^65^. Still, to improve the PL and diminish the lifetime of DCJTB in a substrate, endure significant challenges for its commercialization application. While several types of research have demonstrated that a range of metallic nano- and microstructures can make using ferroelectric lithography^61,66^, few studies have applied such structures to simultaneously enhance the PL intensity and reduce the lifetime of the DCJTB in a substrate. Unlike the substrates mentioned above, this paper proposed a PL-active substrate with a high PL intensity and a short PL’s lifetime of the DCJTB depending on the substrate’s annealing time. We use an inexpensive and easily controlled method to fabricate plasmon-active PPE LiNbO_3_ templates and verify the functionality of the substrate by depositing AgNPs onto the substrates. Furthermore, the chemical patterning used by the proposed procedure offers the capability to manipulate the EM waves in the vicinity of LiNbO_3_ crystals’ surface. Thus, we can simultaneously achieve the remarkable enhanced PL intensity and short PL’s lifetime of DCJTB in the fabricated plasmon-enhanced substrate system. To understand the mechanism, we employ the three-dimensional (3-D) finite element method (FEM) on the dependence of EM wave distribution. The proposed substrate results from surface plasmon resonance (SPR), gap plasmon resonance (GPR), and the confined local EM wave. The substantial plasmonic enhancement makes the fabricated PPE LiNbO_3_ substrate a promising candidate for function as a PL-active substrate.

## Fabrication procedure

**Figure 1:**
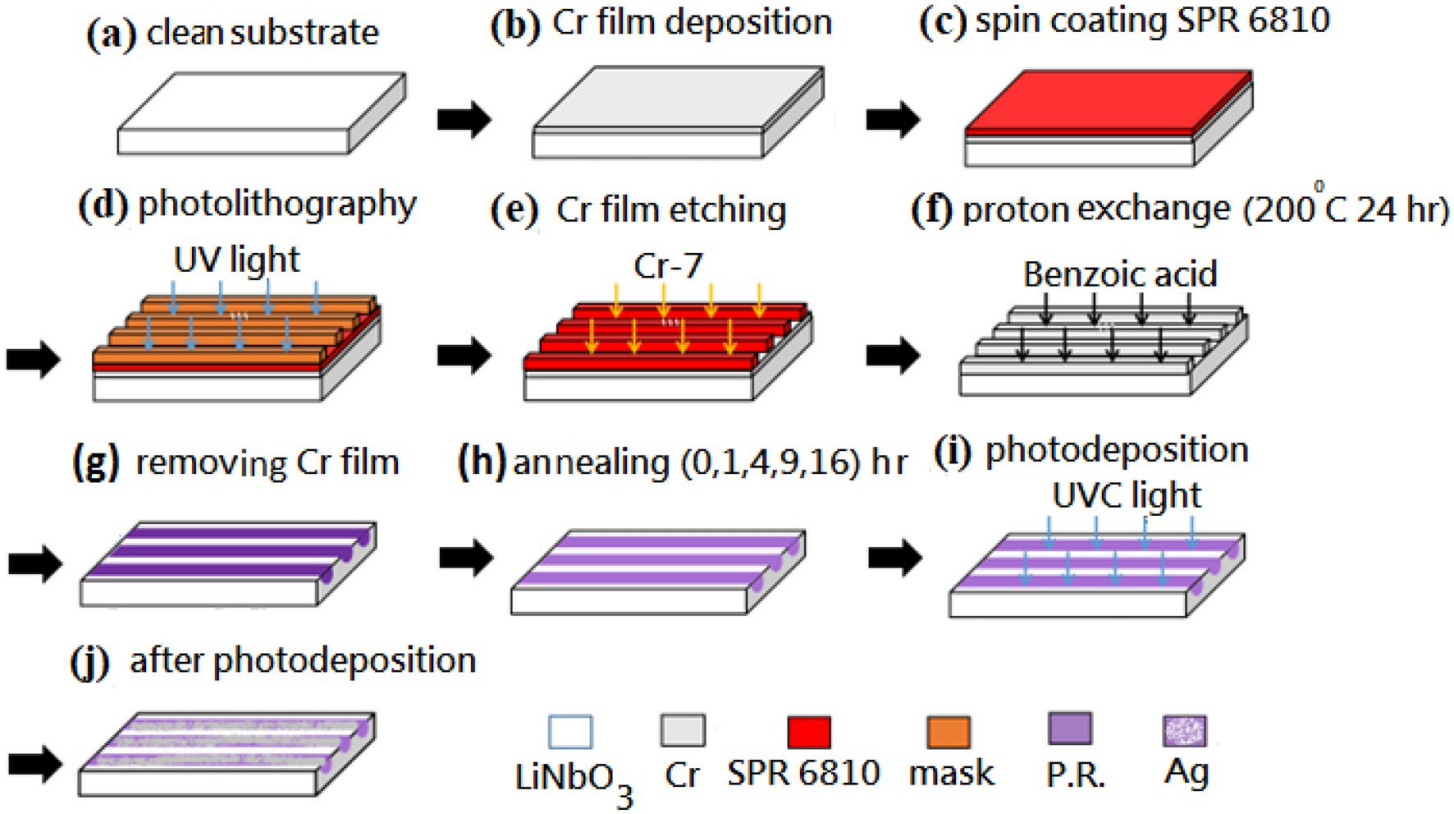
Schematic illustrations of the fabrication procedures of a periodically proton-exchanged (PPE) LiNbO_3_ substrate, (**a**) clean substrate, (**b**) Cr film deposition, (**c**) spin coating SPR 6810, (**d**) photolithography, (**e**) Cr film etching, (**f**) photon exchange (200 °C**,** 24 h), (**g**) removing Cr film, (**h**) annealing (0, 1, 4, 9, 16) h, (**i**) photodeposition and (**j**) after photodeposition.

The PPE LiNbO_3_ shows a lesser density of defects in the vicinity of its surface, which leads to remarkably external screening and nearly flat bands close to the surface^43,55^. PPE LiNbO_3_ substrate can fabricate by electric poling. The LiNbO_3_ single crystal wafer is first to cut perpendicular to the crystallographic c-axis and polished^67^. This paper reports on the main procedures in fabricating flat PPE LiNbO_3_ substrates and the assembly of AgNPs onto the + z domain surfaces of LiNbO_3_. We utilize the nanoscale patterned consistent LiNbO_3_ as the deposition substrates. Figure 1a–j depict the PPE LiNbO_3_ substrate's fabrication procedures. The z-cut with the dimension of 0.8 cm × 0.8 cm × 1 mm of ferroelectric substrates (optical grade, congruent LiNbO_3_ substrates, Crystal Technology Inc.) was adopted to fabricate the PPE LiNbO_3_ substrates. Firstly, the substrate was cleaned using acetone and methanol in an ultrasonic bath for 20 min and then heated on a hot plate at 120 °C for 3 min (Fig. 1a). Subsequently, we deposited a 240 nm-thick Chromium (Cr) film (which uses as a proton exchanged mask) on the substrate surface with a 40 W RF magnetron sputtering under the pressure of 0.6 Pa and Ar flux of 30 sccm for 30 min (Fig. 1b). An SPR6810 photoresist layer was then spin-coated onto the cleaned substrate surface (Fig. 1c). The resulting substrate was heated on a hot plate at 95 °C for 1.5 min. We covered the LiNbO_3_ substrate surface with a periodic Cr-mask patterned via photomask aligner and D-35 developer solution for the photolithography process. An ultraviolet (UV) light with an incident wavelength of 254 nm impinged the substrate for 20 s. We reduced the Cr film's photoresist stripes after heating by a hot plate at 140 °C for 3 min (Fig. 1d). The substrates were then put in an oven at 120 °C for 30 min. After that, we removed the Cr film (which was uncovered by the photoresist mask) using an etching solution and then removed the photoresist using acetone. After photolithography and wet etching, the formed Cr film used as the proton exchanged mask has an opening width of 5.0 μm and a period of 10.0 μm. The Cr mask formed by PPE LiNbO_3_ substrate was then obtained (Fig. 1e). Subsequently, the PPE LiNbO_3_ substrate was immersed in a proton-rich benzoic acid melt at 200 °C for 24 h (Fig. 1f). After that, we cleaned the PPE LiNbO_3_ substrate and removed the patterned Cr film (Fig. 1g). Edwin et al. have found that the cause of annealing on the surface index change and the waveguide depth increase follows a power-law relationship^68^. Therefore, the substrates were annealed at 240 °C for 1 h, 4 h, 9 h, and 16 h for comparison (Fig. 1h). We cleaned the PPE LiNbO_3_ substrates, and then AgNPs deposition was carried out by dripping the 10^–2^ M aqueous solution of AgNO_3_ on the domain patterned LiNbO_3_ crystal surface under UV irradiation (with the incident wavelength of 254 nm) for 3 min at 2 cm (Fig. 1i). When the LiNbO_3_ surface is covered with a metal salt solution such as AgNO_3_ and irradiated by a UV light, the electrons can be quickly induced and migrated toward the positive direction since the band bending energy. The photoelectrons migrating to the substrate surface reduce the Ag ions in the solution to form the AgNPs layer. After UV irradiation, the substrate surface was rinsed with distilled water and then sprayed with nitrogen gas to dry it. After photodeposition, we obtained the fabricated PPE LiNbO_3_ substrates (Fig. 1j).

**Figure 2:**
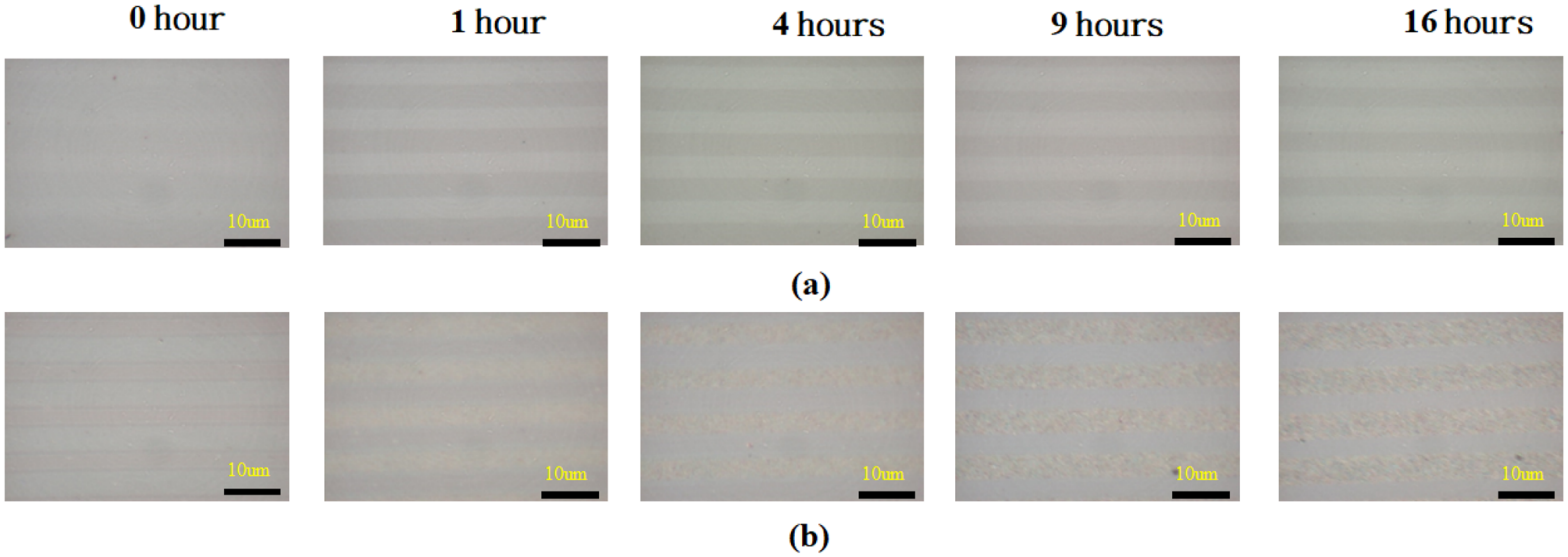
Optical microscope (OM) images of the PPE LiNbO_3_ substrates with the different annealing time of 0-h, 1 h, 4 h, 9 h, and 16 h, (**a**) before photoreduction and (**b**) after photoreduction.

Figure 2 depicts the optical microscope (Carl Zeiss Axiolab) images of the PPE LiNbO_3_ substrates with the different annealing times of 0-h, 1 h, 4 h, 9 h, and 16 h before photoreduction (Fig. 2a) and after photoreduction (Fig. 2b). These optical microscope images reveal the brilliant lines represent the proton exchange regions. In the annealing procedure, the protons can diffuse along with both the out of plane and in-plane, respectively^54^. Therefore, the line width is widened as the annealing time (*t*_a_) increases for *t*_a_ = 0 h and 4 h and shows the unapparent change for *t*_a_ = 9 h and 16 h.

**Figure 3:**
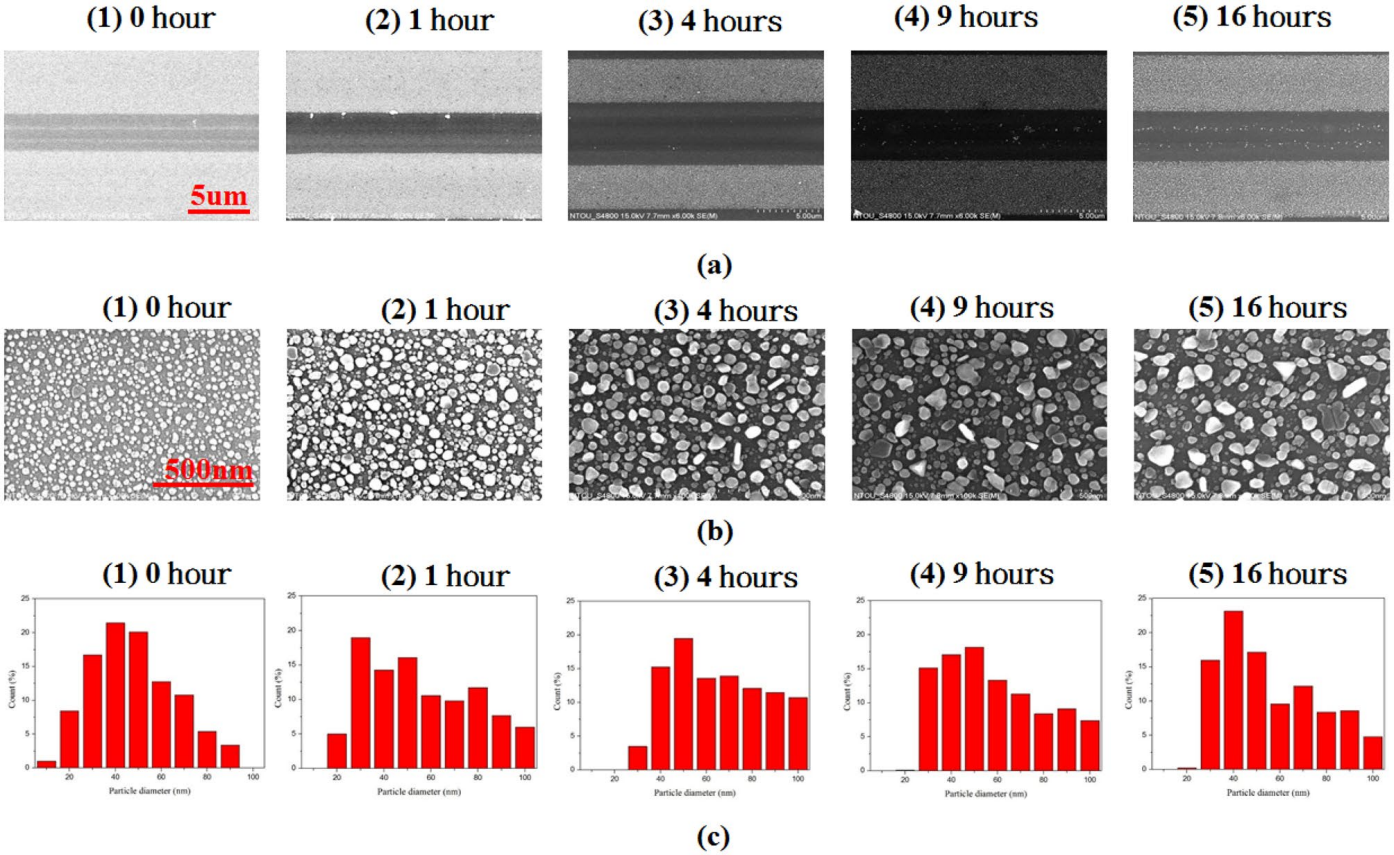
Scanning electron microscopy (SEM) images of photoreduced AgNPs using PPE LiNbO_3_ substrates made with different annealing time, (**a**) with the magnification of 6 K and (**b**) with the magnification of 100 K, respectively. (**c**) Size-distribution histogram of the fabricated PPE LiNbO_3_ substrates under different annealing times of 0, 1, 4, 9, and 16 h.

Figure 3a,b show the SEM images of photoreduced AgNPs using PPE LiNbO_3_ substrates made with various annealing time. As observed, the AgNPs can form in the proton exchanged zone and show a different width of demarcation under variant annealing times. Although the distribution of AgNPs also grows into dense with the increase of annealing time and the gaps of AgNPs become small, the size of AgNPs is approximately similar when *t*_a_ = 9 and 16 h (Fig. 3b4,b5). For the substrate without annealing treatment (*t*_a_ = 0 h), the number of AgNPs is not many, and most of them have a small size (see Fig. 3a1,b1). In Fig. 3a, the two broad lines on the proton exchanged area’s edges relate to the areas with larger AgNPs formed by the edge enhancement of the electrostatic field. As the annealing time increased (see Fig. 3a2–a5,b2–b5), the AgNPs’ size enlarges, and the number of large-size AgNPs grows into a larger area with the larger AgNPs shifting toward the proton exchanged area’s center^54^. This phenomenon demonstrates that the annealing process influences the size of the proton exchanged mask^69^.

**Figure 4:**
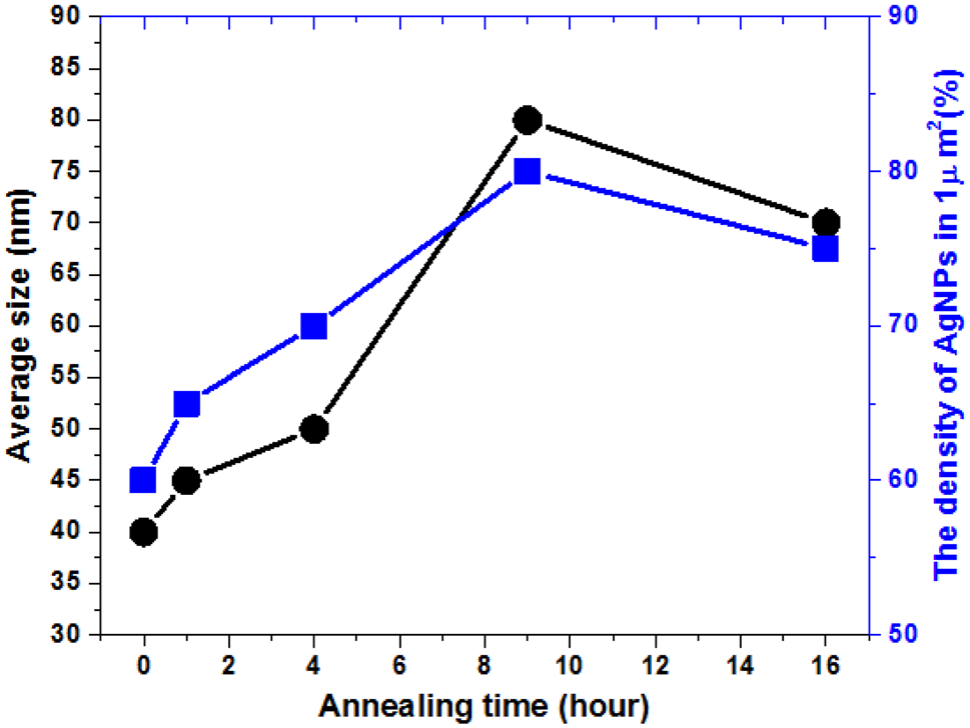
Average size and the density of AgNPs (in 1 μm^2^) as a function of annealing time (hour).

Figure 3c depicts the size-distribution histogram of the fabricated PPE LiNbO_3_ substrates under different annealing times. It can expect that the PPE LiNbO_3_ substrate without annealing treatment (*t*_a_ = 0 h) generates smaller AgNPs in which the size does not reach 100 nm (see Fig. 3c1). As the annealing time increases, the electrostatic field enhanced by the ferroelectric PPE LiNbO_3_ substrate increases photoelectrons and Ag ions, creating more large-size AgNPs^54,66,69^. In the proposed plasmonic system, the absorption peak of localized surface plasmon resonance depends on the gap between adjacent AgNPs, the size and shape of the AgNPs. Among them, the gap between adjacent AgNPs will dominate the plasmonic effect in the fabricated substrates, and this effect was demonstrated by COMSOL Multiphysics simulations (see Fig. 8). We measured the size of AgNPs based on SEM images and histograms. The average diameter of AgNPs is around 45.60 nm, 55.40 nm, 58.39 nm, 63.00 nm, and 57.30 nm for different annealing times of 0, 1, 4, 9, and 16 h, respectively. Note that the annealing time with 9 h has the largest average size of AgNPs and more AgNPs ranging in 30 ~ 100 nm than the other annealing time, exposing the larger surface of AgNPs in the same area. These results from Fig. 3b,c have correlated with the annealing time. The edge enhancement effect, which possesses more dipole effects (i.e., more positive–negative charge pairs on the Ag surface^70–74^), leads to the EM wave enhancement and helps form a different size of AgNPs depending on the various annealing time. It indicates that the ferroelectric PPE LiNbO_3_ substrate's EM wave enhancement can effectively increase the growth rate of AgNPs and facilitate the formation of large-size AgNPs. Figure 4 shows both the average size and the density of AgNPs (in 1 μm^2^) as a function of annealing time (hour). The annealing time with 9 h has the largest average size and the density of AgNPs compared to the other annealing time.

## Measurement results and discussion

**Figure 5:**
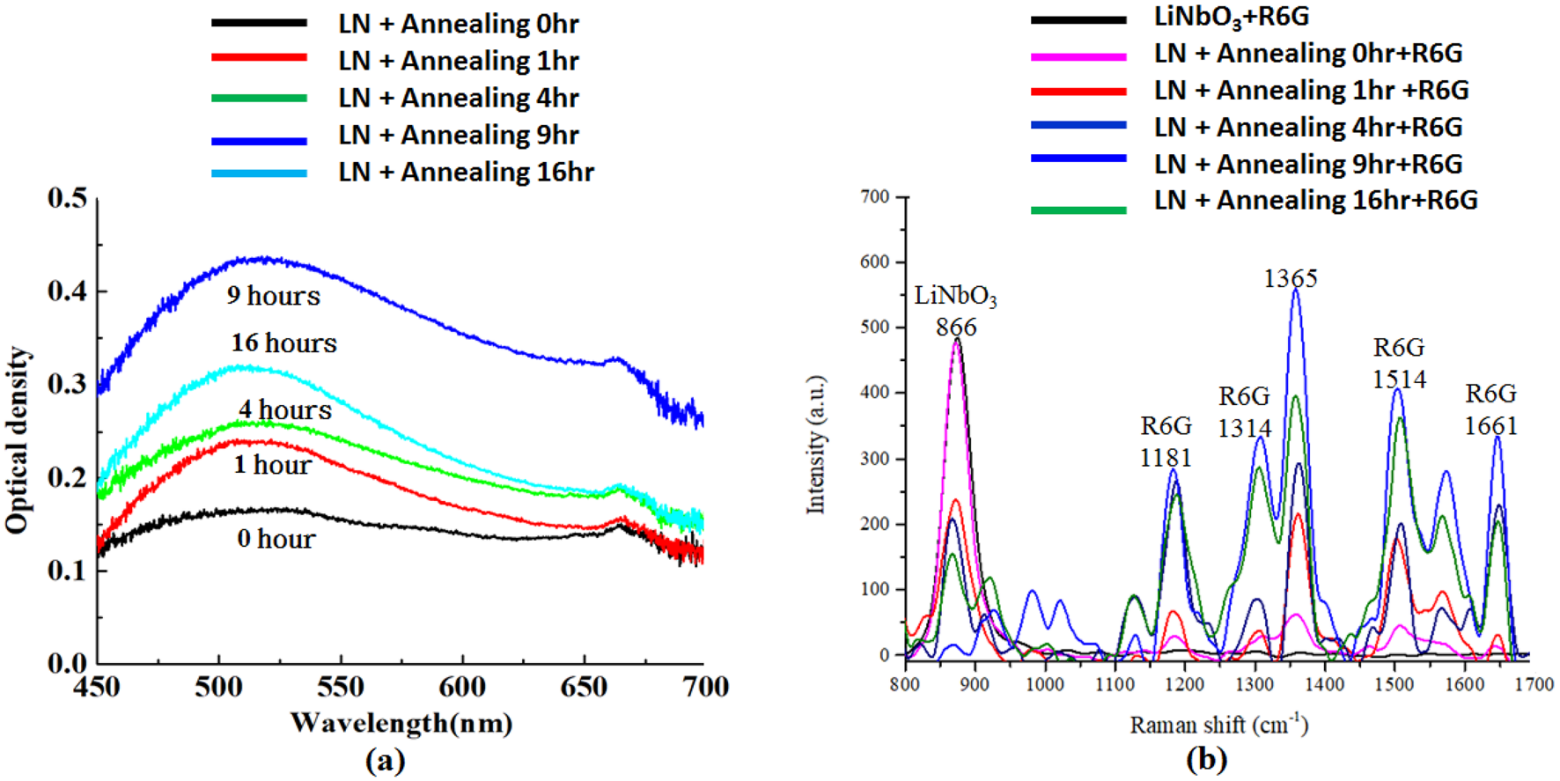
(**a**) Optical density under different annealing times of 0, 1, 4, 9, and 16 h. (**b**) Raman spectra of the rhodamine 6G (R6G) dye on the flat LiNbO_3_ and fabricated PPE LiNbO_3_ substrates under different annealing times of 0, 1, 4, 9, and 16 h.

Optical density, denoted as log_10_ (1/T), increases with larger absorption excited by the localized SPR on the AgNPs’ surface. Figure 5a shows the optical density spectra of the fabricated substrate under various annealing time of 0, 1, 4, 9, and 16 h, respectively. Two prominent optical density peaks for all cases occur around λ = 520 nm and λ = 665 nm, corresponding to two SPR modes of the fabricated PPE LiNbO_3_ substrate. The first peak with the maximal optical density at 520 nm is near the measurement's excitation wavelength. The maximal peak value of optical density is *t*_a_ = 9 h. The magnitude of optical density is strongly related to the localized SPR effect generated from the interaction between the incident EM wave and the AgNPs on the fabricated PPE LiNbO_3_ substrate. The wavelength corresponds to the optical density peak of the fabricated substrate treated with different annealing times of 0, 1, 4, 9, and 16 h. In Fig. 5a, the OD spectra do not show the size-dependent shift upon changing the annealing time since the average size of AgNPs shows a minor difference, and the size and distribution of AgNPs are not uniform. Therefore, the OD spectra reveal a broadband profile. Note that the intensity variation in the optical density's tendency with the annealing time is directly proportional to the AgNPs density, as mentioned above. This phenomenon indicates that the fabricated substrate with *t*_a_ = 9 h shows the most plasmonic effect on the AgNPs’ surfaces compared to other annealing times. The case of *t*_a_ = 0 h (i.e., non-annealed substrate) shows the smallest optical absorption because the smaller and lower density of AgNPs can produce on the fabricated substrate surface. Figure 5a shows that the density and distribution of AgNPs in the fabricated substrate greatly influence the plasmonic effect and optical absorption. Such remarkable enhancement is excited by plasmonic coupling between the AgNPs and incident EM wave. This result is direct evidence of AgNPs contributing to the fabricated PPE LiNbO_3_ substrate's optical density performance. Furthermore, it is illustrated through the optical density spectrum that the AgNPs can confine surface plasmons if the gap between the adjacent AgNPs is sufficiently small.

The assembly of metallic nanostructures can further functionalize with a target molecule from detecting the Raman signal. If a target molecule is under the hot spot of the EM wave, it will generate a relatively large Raman signal from the target molecule. We used a laser confocal Raman microspectrometer (equipped with a 532 nm laser) to inspect the EM wave scattering of a target molecule. All measurements utilized a 20 × /NA0.4 objective, an R500-2000 line/mm grating, a data integration time of 5 s, and a small laser power set at 1 mW. Each substrate was subjected to ten measurements at different spots to obtain an average Raman signal. We used a representative organic analyte, rhodamine 6G (R6G), with 10^–5^ M as the target molecule for Raman signal measurement. R6G has well-established vibrational features and extensive surface-enhanced scattering properties. Figure 5b depicts the Raman spectra of the R6G dye on the flat LiNbO_3_ and the fabricated PPE LiNbO_3_ substrates produced with different annealing times of 0, 1, 4, 9, and 16 h. The various annealing time of the substrates exhibits tunable PL activities. It can observe that six distinct Raman intensity peaks appeared in the range of 800–1700 cm^−1^, which can contribute by the various vibration modes of the R6G molecules. The intensities of the R6G’s Raman peaks from the fabricated substrates are related to the interaction between the vibration modes and the localized surface plasmon mode. Such enhancement in Raman signals can attribute to the plasmonic effect arising from the surface plasmon on AgNPs, the gap plasmon enhancement among AgNPs, and the charges transfer between the fabricated PPE LiNbO_3_ substrate and R6G. This result implies that one can get Raman signal enhancements using the most favorable photoreduced MNP nanostructure, i.e., the fabricated PPE LiNbO_3_ substrates^54^. As discussed in Fig. 5a, we can tune the plasmonic effect by the different annealing times. As the annealing time changes, it can also alter the Raman signals' corresponding plasmonic response. Note that the optimal value of annealing time is *t*_a_ = 9 h. It leads to enhanced E-field localization in the gap and edge regions among the AgNPs and the surface of the AgNPs. These effects are beneficial to improve the Raman signals. In Fig. 5b, the Raman peaks of the R6G dye occurs at the wavenumbers of 1181 cm^−1^, 1314 cm^−1^, 1365 cm^−1^, 1514 cm^−1^and 1661 cm^−1^, and it does not appear in the case of flat LiNbO_3_, which Raman peak is only happened at 866 cm^−1^ and exhibits the highest one compared to other annealing times. In these AgNPs-assisted plasmon-enhanced substrates, the weak Raman signal can collect at the flat LiNbO_3_ and *t*_a_ = 0, 1, and 4 h with the smaller Raman peaks at 1181 cm^−1^, 1314 cm^−1^and 1661 cm^−1^, whereas stronger Raman signals can achieve at *t*_a_ = 9 and 16 h with remarkably Raman peaks. The Raman peaks at 1365, and 1514 cm^−1^ demonstrated the generation of R6G dye produced by the fabricated plasmon-enhanced substrates when *t*_a_ = 4, 9, and 16 h. The Raman signal intensity at 1365 cm^−1^ on the fabricated PPE LiNbO_3_ substrate can assign to the aromatic C–C stretching of R6G molecule^75^. Figure 5b demonstrates that the Raman signal intensities increase with the increasing annealing time. The maximal intensity peak occurs at *t*_a_ = 9 h, with the maximal plasmon-enhanced effect compared to the other annealing time. The plasmon-enhanced phenomenon is attributed to the most substantial electric field enhancement results from many closely adjacent, large-diameter AgNPs, as indicated in Fig. 3b4, with high-density inter-particle gaps in subwavelength scale, and thus more gap plasmon resonance effect. It is worth noting that the highest measured Raman peak of *t*_a_ = 9 h at the wavenumber of 1365 cm^−1^ is enhanced 10.36 times compared to *t*_a_ = 0 h (i.e., un-annealed substrate), exhibiting the noticeably improved sensitivity. The Raman intensity spectra obtained from the R6G molecules adsorbed on the various PPE LiNbO_3_ substrates can demonstrate the enhanced magnitude and scattering of the EM waves in the fabricated plasmon-enhanced substrates (i.e., the bright spots arising from the plasmonic effect).

**Figure 6:**
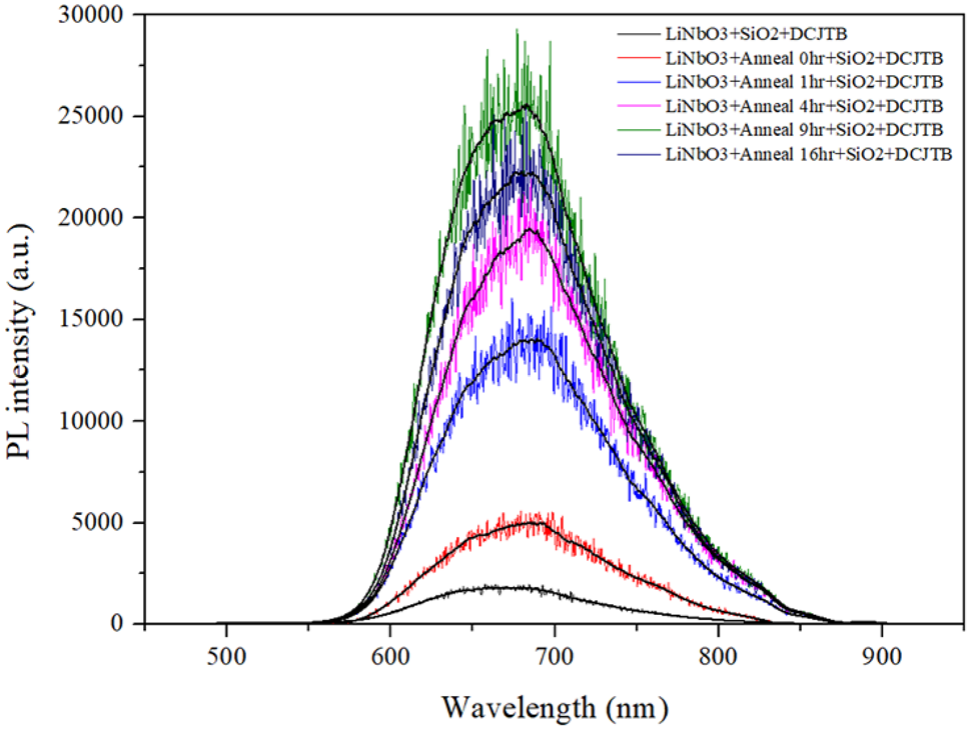
Photoluminescence (PL) of the flat LiNbO_3_ substrate and the fabricated PPE LiNbO_3_ substrates under different annealing times of 0, 1, 4, 9, and 16 h.

For obtaining the PL spectra, 375 nm CW laser was employed to excite the DCJTB emission, whose absorption is around 510 nm^76^. Besides, the PL signal can be enhanced when the resonant mode of plasmonic nanoparticles is spectrally overlapped with either the absorption wavelength or the emission position of the photon emitters^77,78^. The excitation laser is away from the peak wavelength of the plasmonic modes in nanoparticles. However, the spectral overlapping between the plasmonic absorption and emitted photon energy can be obtained due to the relatively broad absorption spectrum (see Figs. 5a and 6). As a result, the observed PL enhancements are initially from the near-field coupling between the DCJTB and Ag nanoparticles. Note that the absorption peak at ~ 670 nm is another plasmonic mode, which occurs in all cases in Fig. 5a and contributes to enhancing the PL signal. The absorption at ~ 670 nm possesses almost equal intensity in all cases, further verifying that the sample-dependent PL enhancement is mainly from the broad plasmonic absorption centered at ~ 510 nm.

The localized SPR wavelength of AgNPs at 510 nm can couple well with the DCJTB molecular, thus leading to the plasmonic enhancement of the DCJTB emission. Figure 6 shows the PL measurement results of the flat LiNbO_3_ and the fabricated PPE LiNbO_3_ substrates under various annealing times of 0, 1, 4, 9, and 16 h. A CW laser light of 375 nm wavelength with a 0.4 mW power and an integration time of 1 s is transmitted through a mirror into an inverted microscope and focused onto the substrate via an objective lens with 0.9 NA and 100 × magnification. To prevent energy transfer quenching, we insert a SiO_2_ buffer between the substrate and the DCJTB fluorescent dye layer. The thickness of the SiO_2_ buffer layer and DCJTB is 10 nm and 75 nm, respectively, as discussed in our previous literature^35,79^. In Fig. 6, all the spectral profiles had similar shapes. The exposed area of the AgNPs surface can generate PL intensity enhancement because the increased PL intensity could be attributed to DCJTB molecules' interactions with the AgNPs surface, offering a plasmon resonance. A noticeable difference in PL intensity in Fig. 6 can observe under different annealing times. The PL intensity of the case of *t*_a_ = 0, 1, 4, 9, and 16 h can reach a maximum value of 4699, 13,801, 18,343, 23,933, and 21,086, respectively, which are 2.69, 7.9, 10.5, 13.7, and 12.07 times the intensity compared to the flat LiNbO_3_ substrate.

Subsequently, we measured the lifetime of the dye molecules (DCJTB with 75 nm) for flat LiNbO_3_ substrate and the fabricated PPE LiNbO_3_ substrates under different annealing times of 0, 1, 4, 9, and 16 h. The lifetime measurement method is reported in the previous works^35,62,79^. We used a laser beam (λ = 375 nm, power = 0.4 W) focused onto the substrate and performed a 2D XY-scan of 50 × 50 μm^2^ area with simultaneous time-resolved PL (TRPL) measurements and recorded the light intensities of each spot^20,80^. A biexponential function, I _PL_(t) = ∑A_*i*_ exp(− t/τ_*i*_) + y_0_, is used to fit the measured PL decay curves. Where τ_*i*_, A_*i*_, and y_0_ denote decay time, amplitude, and background intensity, respectively^35,62,81–83^. For simplicity, the measured PL decay lifetime is given by 1/ τ_TRPL_ = 1/τ_1_ + 1/τ_2_^20^.

**Figure 7:**
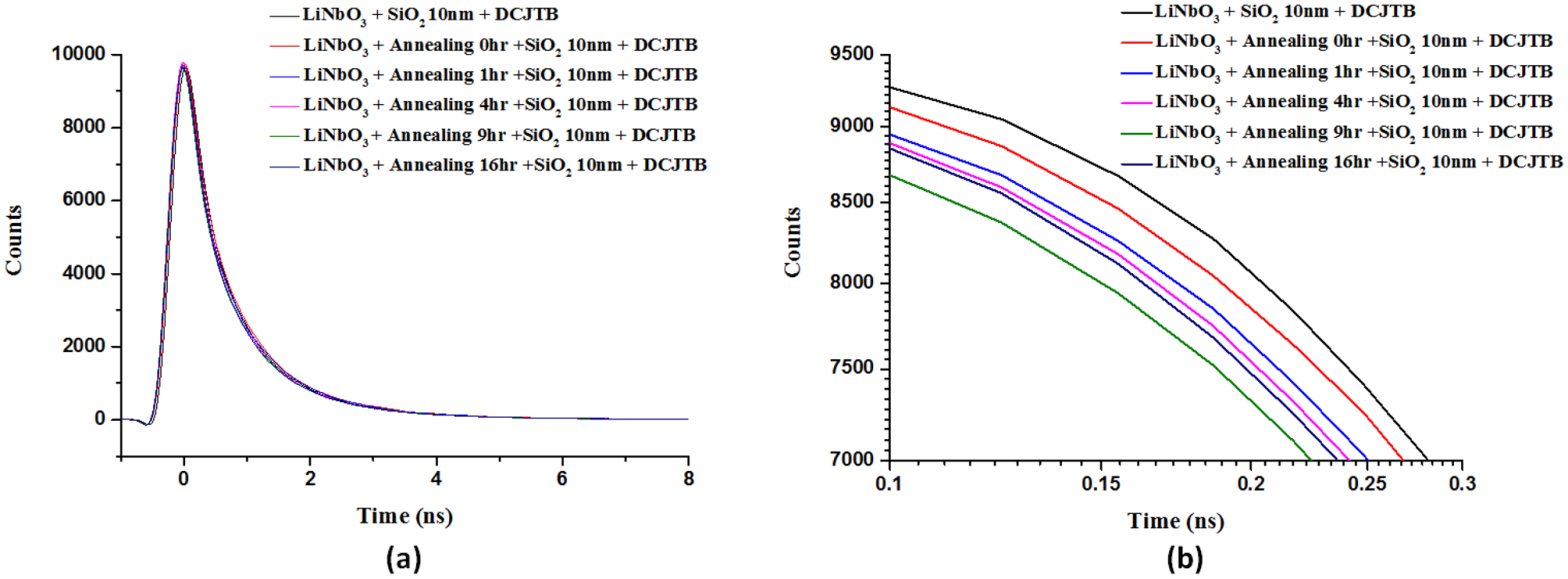
(**a**) Lifetime of the dye molecules (DCJTB with 75 nm) for the flat LiNbO_3_ substrate and the fabricated PPE LiNbO_3_ substrates under different annealing times of 0, 1, 4, 9, and 16 h, respectively. (**b**) Enlarged diagram of (**a**) in the time range of 0.1–0.3 ns.

In Fig. 7, the dye molecules' decay rates on the fabricated PPE LiNbO_3_ substrates treated with annealing time are faster than the corresponding one without annealing and the flat LiNbO_3_ substrate. By combining all the luminescence spectrum areas and comparing the intensities from DCJTB on the PPE LiNbO_3_ substrates, the lifetime of 1.19 ns can be achieved under the annealing time, *t*_a_ = 9 h. The rest of the lifetime for DCJTB substrate, *t*_a_ = 0, 1, 4, and 16 h are 1.36, 1.35, 1.32, 1.30, and 1.26 ns, respectively. The lifetime at *t*_a_ = 9 h is reduced by 12.50% (from 1.36 to 1.19) compared to DCJTB substrate. These results can be elucidated by the strong EM wave coupling when the substrate nanostructure's plasmon resonance overlaps with the molecules' absorption band^35^.

**Figure 8:**
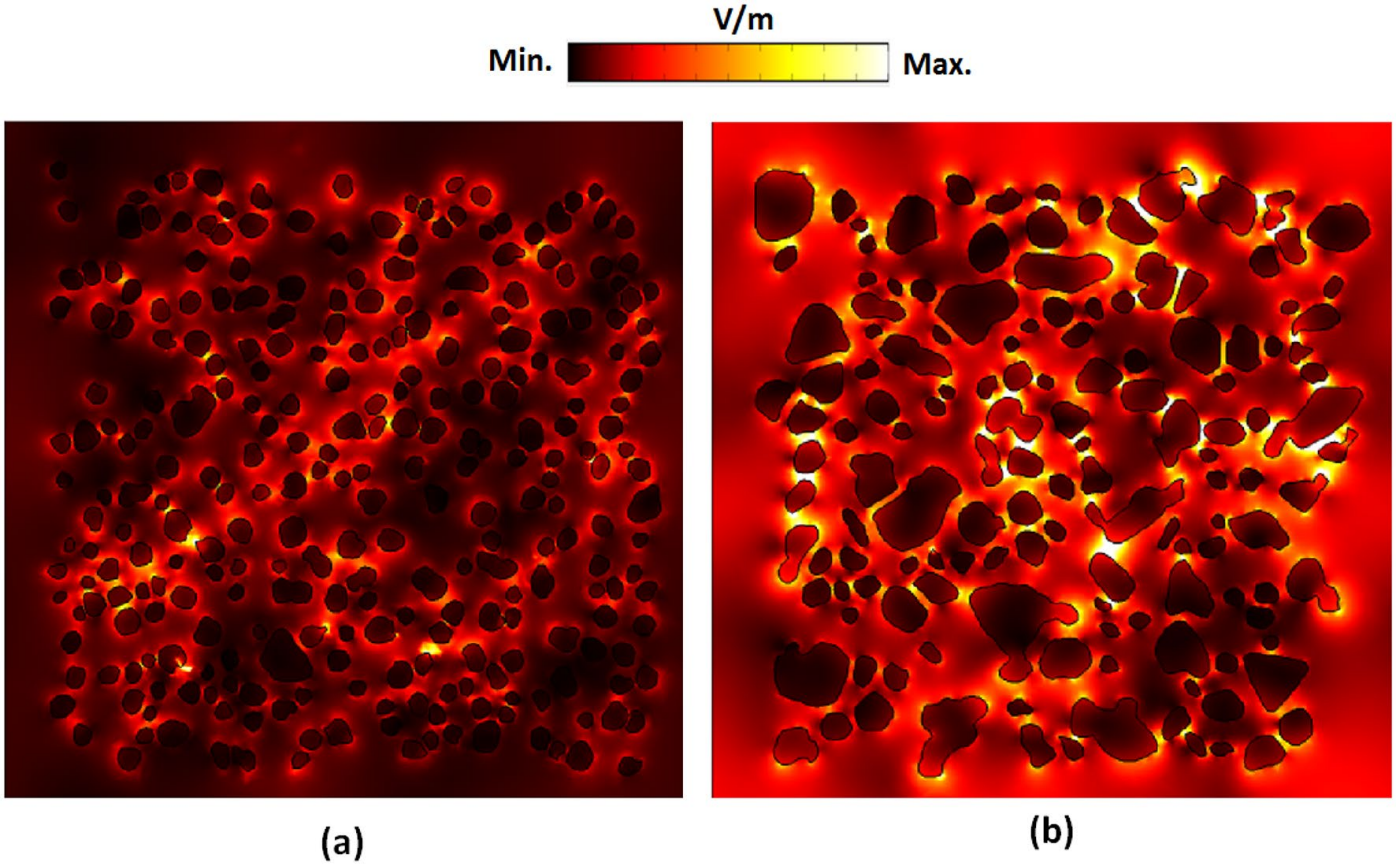
Spatial electric field distribution with an incident wavelength of 650 nm on the fabricated PPE LiNbO_3_ substrate surface under (**a**) *t*_a_ = 0 h and (**b**) *t*_a_ = 9 h, respectively.

We used COMSOL Multiphysics simulations to understand the mechanism of electric field distribution on the surface of the proposed substrate. The simulations used a 3-D model with perfectly matched layer boundary conditions^84^. Since the case of *t*_a_ = 9 h exhibits the most robust fluorescence than the other annealing time, we compare the two cases, *t*_a_ = 9 h and *t*_a_ = 0 h, in our simulation models. The sizes of simulation models are 875 nm(*x*) × 875 nm(*y*) × 60 nm(*z*) for the case of *t*_a_ = 9 h and 875 nm(*x*) × 875 nm(*y*) × 40 nm(*z*) for the case of *t*_a_ = 0 h, respectively. Circularly polarized light with an incident wavelength of 650 nm and electric field intensity of E_0_ = 1 V/m is launched normally from the top of the substrates. The permittivity data of Ag can obtain from Refs.^85^. The plasmonic effect can mainly contribute by enhancing of the localized surface plasmon acting on the nanostructure^86–89^. Figure 8a,b depict the simulated spatial electric field distribution induced by the incident light of 650 nm impinging on the fabricated PPE LiNbO_3_ substrate with *t*_a_ = 0 h and *t*_a_ = 9 h, respectively. The localized electric field in the vicinity of the AgNPs with the larger diameter and smaller gap distance under *t*_a_ = 9 h is more vital than that of a smaller one under *t*_a_ = 0 h. It is consistent with the above PPE's experimental results (Fig. 3c1,c4), which possess the maximum number of AgNPs in the diameter range of 30 ~ 100 nm, showing the most substantial absorption at 650 nm. The simulation result also displays a strong localized electric field in the gaps among AgNPs. The significant electric field enhancement around the large-diameter AgNPs and the small gap between AgNPs suggests the leading cause of the PL enhancement in the fabricated substrate. These results are relevant to PL-active substrates' design, and we can expect a larger PL with plasmon enhancement.

## Conclusions

To summarize, we have demonstrated a reliable and relatively easy method to construct a plasmon-enhanced photoluminescence substrate with a shortened lifetime. We studied the DCJTB’s PL, and lifetime depending on the substrate's annealing time. The chemical patterning offers the capability to manipulate the EM waves near the surface of lithium niobate substrate, and these engineered fields can apply to fabricate the assembly of metallic nanostructures, which have a PL-active function. The PL intensities and shortened lifetimes of DCJTB in the fabricated substrate confirmed the presence of enhanced local EM waves by SPR and GPR modes, which are strongly confined on the substrate’s surfaces and in the AgNPs’ gaps. In the AgNPs-assisted photoreduction, enhancing the electrostatic field facilitates large-diameter AgNPs, and can achieve intense localized EM wave action by annealing time. 3D-FEM simulations demonstrated that the confined electric field around the AgNPs with larger size under *t*_a_ = 9 h are more vital than weaker fields surrounding the smaller AgNPs under *t*_a_ = 0 h. Compared with the un-annealed substrate, the fabricated PPE LiNbO_3_ substrates with ferroelectric AgNPs can enhance the PL intensities of DCJTB by 13.70 times and reduce the PL’s lifetime by 12.50% (from 1.36 to 1.19) at *t*_a_ = 9 h, respectively. The fabricated ferroelectric PPE LiNbO_3_ substrates with the vigorous photoreduction characteristic promise versatile PL applications for practical optical device applications.

## Data Availability

The datasets generated and analyzed during the current study are not publicly available due to compelling reasons why data are not public but are available from the corresponding author on reasonable request.
